# “If it weren’t for my traditional healer, I would be dead”: Engaging traditional healers to support people living with HIV in rural Mozambique

**DOI:** 10.1371/journal.pone.0270565

**Published:** 2022-06-28

**Authors:** Carolyn M. Audet, Mariah Pettapiece-Phillips, Yuqi Tian, Bryan E. Shepherd, Sten H. Vermund, Jose Salato

**Affiliations:** 1 Department of Health Policy, Vanderbilt University, Nashville, TN, United States of America; 2 Department of Biostatistics, Vanderbilt University, Nashville, TN, United States of America; 3 School of Public Health, Yale University, New Haven, CT, United States of America; 4 Friends in Global Health, Quelimane, Mozambique; Centre for Sexual Health & HIV/AIDS Research (CeSHHAR)R, ZIMBABWE

## Abstract

Across rural sub-Saharan Africa, people living with HIV (PLHIV) commonly seek out treatment from traditional healers. We report on the clinical outcomes of a community health worker intervention adapted for traditional healers with insight into our results from qualitative interviews. We employed a pre-post intervention study design and used sequential mixed methods to assess the impact of a traditional healer support worker intervention in Zambézia province, Mozambique. After receiving a positive test result, 276 participants who were newly enrolled in HIV treatment and were interested in receiving home-based support from a traditional healer were recruited into the study. Those who enrolled from February 2016 to August 2016 received standard of care services, while those who enrolled from June 2017 to May 2018 received support from a traditional healer. We conducted interviews among healers and participants to gain insight into fidelity of study activities, barriers to support, and program improvement. Medication possession ratio at home (based on pharmacy pick-up dates) was not significantly different between pre- and post-intervention participants (0.80 in the pre-intervention group compared to 0.79 in the post-intervention group; p = 0.96). Participants reported receiving educational and psychosocial support from healers. Healers adapted their support protocol to initiate directly observed therapy among participants with poor adherence. Traditional healers can provide community-based psychosocial support, education, directly observed therapy, and disclosure assistance for PLHIV. Multiple factors may hinder patients’ desire and ability to remain adherent to treatment, including poverty, confusion about medication side effects, and frustration with wait times at the health facility.

## Introduction

Antiretroviral therapy (ART), when taken regularly, effectively suppresses HIV viral replication among persons living with HIV (PLHIV) [1–3]. People with delayed, disrupted or discontinued ART experience rapid disease progression, develop resistance to ART, and remain more likely to transmit the virus during sexual activity [4–6]. Though widespread implementation of “test and treat” has improved ART coverage [7–9], poor adherence and treatment interruptions are common, particularly among those living in communities with low socio-economic status where poorly-functioning health systems (medication stock outs, poor quality care, low patient satisfaction) [10–15], sociocultural norms and beliefs (HIV stigma, mistrust in health system) [10,11,16–24], economic constraints (food insecurity, lack of transportation) [12,19,24–27], as well as family and individual barriers (HIV knowledge, preference for traditional medicine) [19,20,25,27,28] undermine access and willingness to remain in treatment.

The Mozambique Ministry of Health has adopted participant-centered ART delivery models [29] designed to reduce transportation costs and time waiting at the health facility, increase social support, and provide counseling to someone who recently missed an appointment. Despite these interventions, only 71% of participants remain engaged (defined as one medication pick-up within the past 59 days) one year after enrollment in 2019 [30]. However, when applying more conservative definitions (e.g. medication possession ratio), estimated adherence to medication is substantially lower [31,32]. Drug shortages and dosing errors represent additional challenges [33].

Additional evidence-based interventions to address barriers to medication retention include community ART distribution points [34,35], adherence clubs [31,35–38], spaced refill visits [39,40], and employing Community Health Workers (CHWs) for ART provision and education [41–44]. When implemented with fidelity, CHW programs have shown to increase patient adherence to ART, leading to improved viral suppression [45]. One reason these interventions are successful is that they create a more equitable health care experience: CHWs are more likely to be of the same ethnic, racial, and socioeconomic status as their patients [41,46,47], potentially eliminating some of the bias experienced by patients at the health facility.

In rural Mozambique, CHWs have struggled to effectively implement interventions due to a lack of transport, insufficient training programs, poor integration into the clinical system, and frequent turnover among volunteer staff [43,44,48]. To overcome these challenges, we sought to employ traditional healers to support PLHIV; across rural sub-Saharan Africa (SSA), people living with HIV commonly seek out treatment from traditional healers [49]. Healers provide care that is socially acceptable, are well-respected, and already have an existing relationship with members of the community [23,50]. Moreover, healers have been shown to influence patients’ attitudes and behaviors towards HIV care utilization [24,51]. To capitalize on these existing health providers, we adapted a CHW intervention for implementation by traditional healers. Engaging traditional healers to provide education, social support, and counseling overcomes many common logistical issues, given that healers live in the same communities as their patients and have a history of providing medical services to their constituents. However, the employment of individuals who have historically been ostracized and excluded from the health system is not without risk [52,53]. In this manuscript we report on the clinical outcomes of the trial with insight into our results from qualitative interviews with healers and people living with HIV who participated in the program.

## Materials and methods

### Author positionality

This study was led by a white Canadian (now also American- CMA) and a Black Mozambican researcher (JS). While CMA largely relies on allopathic medicine to treat illness, JS is comfortable with both herbal and allopathic medicine, depending on the illness in question. We both believe that people living with HIV should receive support from whatever source they choose, and traditional healers are one group that have the access to supporting people living with HIV without attracting attention, at least in this part of Mozambique [50]. In addition to our quantitative data, we offer these qualitative findings as only one possible interpretation of our participants’ experiences with the HIV care and treatment system and their traditional healer.

### Study location

Zambézia province is in north-central Mozambique. Zambézia is home to 5.1 million people (2017) and is one of the poorest regions in the world [54]. About half (51%) of adults over 15 are literate [55,56]. The adult HIV prevalence was 15% in 2015, one of the highest in sub-Saharan Africa (SSA) [57,58]. Traditional healers have been engaged in the health system for more than ten years, with a focus on creating formal systems of partnership between healers and local clinics [51,52,59] largely because they were seen as the preferred and trusted partners of people living with HIV [23,60–63].

### Study design

We employed a sequential, mixed methods, pre-post intervention study design to assess the impact of a traditional healer support worker intervention. Participants who enrolled from February 2016 to August 2016 received standard of care services, while those who enrolled from June 2017 to May 2018 received standard of care services plus support from a traditional healer. Interviews with participants were completed with a select number of participants after the completion of the intervention.

### Study population

People living with HIV who had received a new positive HIV test result via the voluntary counseling and testing service unit and enrolled in HIV care and treatment at a health facility in Namacurra from February 2016 to May 2018 were eligible to participate in the study. Pregnant and postpartum women and those under 18 years of age who received testing in other hospital units and people who reported previous enrollment in ART services, were excluded from the study. A study assistant described the study to the participant immediately after their positive test result. Those who were interested in receiving support from a traditional healer were recruited but only those who enrolled in HIV care and treatment services were enrolled (44 individuals expressed interest in healer support but ultimately did not enroll in treatment services). Only those who enrolled after June 2017 were provided healer support. Of those approached, 95% of participants agreed to participate. Among those who reported a reason for refusing to participant all did so because they did not trust traditional healers to support them or because their religion did not allow for them to visit a traditional healer.

#### Standard of care protocol

Participants who enrolled in the study received the standard of care support, including: (1) Counseling sessions at the health facility (held monthly) for the first three months of treatment and every 3 to 6 months after treatment stabilization, (2) ART and other medications at no cost, (3) Semi-annual viral load testing, and (4) Peer support home visits if the participant became lost to follow up.

#### Intervention protocol

The evidence-based intervention was based on the Adherence Support Worker program [53] created in Malawi, originally designed for use by community health workers. We tailored the program for use by traditional healers using the ADAPT-ITT framework [64] in 2017 [51] to allow for participants to select the healer of their choice. In the adapted version, the essential “core” components of the intervention include weekly visits from a traditional healer with participants to deliver (1) education about HIV medication side effects and strategies for overcoming them, (2) counseling to understand barriers to medication adherence and the co-creation of strategies to overcome these challenges, (3) facilitation of HIV status disclosure to family members, and (4) advocacy of participant needs/concerns to health care providers.

We provided traditional healers who were selected for the program two weeks of counseling and clinical training and 6 hours of contact time with local health care providers to facilitate relationship building.

#### Intervention procedures

Trained healers were notified once they were selected by a participant and subsequently organized a mutually agreed upon time and place (via phone or in person if they knew each other) to begin counseling sessions. The healer and participant would continue to meet bi-monthly and at additional times (e.g., to accompany a participant to the health facility or to pick up medications if the participant was too ill to travel) as desired by the participant.

#### Qualitative interviews

We used purposeful sampling to select 30 participants from the post-intervention group for in-depth interviews, with a focus on identifying people with varying levels of medication adherence and those who live both near and far from the health facility. We recruited 23 of those selected (3 were traveling outside of the district and 4 were not found despite 3 attempts to locate them); all who were approached agreed to complete an in-depth interview.

We reached out to 24 traditional healers who provided participant support (2 died during the study); 19 participated in one of 2 group sessions. Among the 5 healers who did not participate, 3 were working and 2 were traveling at the time of our focus groups.

#### Ethics, consent, and permissions

This study was approved by the Vanderbilt University Institutional Review Board (IRB# 150217) and the Zambézia Committee of Bioethics for Health (IRB# 03/DIBS-Z/15). All participants provided informed consent in their preferred language. Interviewers read the consent form aloud to each participant, discussed each section, and requested those who agreed to participate to either sign the form or place a thump print on the form (using an ink pad).

### Data collection

#### Quantitative data

Participants were enrolled between February 2016 and May 2018. Demographic information was collected from all participants at enrollment. Clinical data from each participant were pulled from OpenMRS, an open-source medical record system used in 40 countries [65]. Clinical data included medication pick-ups, viral load results, and clinical visits for 13 months from enrollment date. Mortality was assessed via data from OpenMRS and report from participant families. Our primary outcome, participant medication possession ratio [16,66–69], was calculated as the sum of the number of days a person living with HIV had medication divided by the total number of days of follow-up. Specifically, every participant was given 30, 60, or 90 days to pick up their subsequent supply of medication as indicated in the medical charts. If a participant picked up medication after the indicated date they were considered “non-adherent” for each day after their specific pick-up date. Participants were followed for 13 months from enrollment date (which was the ART initiation date) unless they died during this period. For participants who died, date of death was considered the final date of follow-up. For participants who picked up medication only once at enrollment (28 participants), their final day of medication coverage was the next expected date of medication pickup; unless these participants died, their follow-up was 13 months from ART initiation date. Traditional healers provided demographic information and completed surveys in empathy [70], narcissism [71], and HIV knowledge immediately post-training [72].

#### Qualitative data

The Consolidated Criteria for Reporting Qualitative Studies guided our reporting of qualitative data collection, analysis, and findings [73]. Between July and September 2019, we conducted 23 semi-structured in-depth interviews at the homes of participants and 2 focus groups at a community engagement office in Namacurra District, Mozambique. The in-depth interview guide was informed by Carroll’s Conceptual Framework for Implementation Fidelity [74] and employed open-ended questions which were asked of all participants in the same order in each interview (Appendix 1). Questions were focused on gaining insight into a participant’s experience with study activities, type, timing, and frequency of services provided, and the perceived quality of the healer support. We also elicited suggestions for program improvement. The focus group guide for traditional healers consisted entirely of open-ended questions, designed to gain insights into the fidelity of delivering study activities, barriers to participant support, modifications that were implemented during the study, activities that were perceived as most successful, and suggestions for program improvement.

Two trained qualitative fieldworkers and the study PI conducted one-on-one interviews and focus groups. The two focus groups, held with the traditional healers, were conducted in Portuguese while one-on-one interviews were conducted in the principal local language, Echuabo. The interviewers have received training in qualitative methods while working with several NIH-funded research projects over the past 12 years. The in-depth interview participants had not previously met the interviewers. The traditional healers had previously met the interviewers during a baseline survey in 2017. Interviews were conducted at the home (or preferred location) of each participant while focus groups took place at a community field office for community health support from a local non-governmental organization, Friends in Global Health. Field notes were taken during the interviews and focus groups; after each interview, the interviewers, JS, and CMA would meet to discuss each session to determine if we were reaching data saturation and what additional questions we still had. After being audio-recorded, interviews were transcribed in either Echuabo or Portuguese (depending on the language preference of our participant). Interviews in Echuabo were subsequently translated to Portuguese. Each transcription and translation were cross-referenced to ensure quality. All Portuguese transcriptions were subsequently translated to English by a professional, certified translator and back translated to ensure accuracy. Duration of participant interviews averaged 26 minutes (range 14–155 minutes). Healer focus groups averaged 1 hour and 15 minutes (range 18–132 minutes).

### Analysis plan

#### Quantitative data

Persons living with HIV and traditional healer characteristics are presented as frequencies with percentages or medians with interquartile ranges (IQR). Participant characteristics at treatment initiation were compared across pre- and post-intervention groups using chi-squared and rank sum tests. We compared the medication possession ratio (a continuous variable with range 0–1) within 13 months of ART initiation between pre- and post-intervention groups using the Wilcoxon rank sum test. We use the medication possession ratio, defined as the proportion of days with medication within 13 months, as the response variable to represent medication adherence. We first compared the medication possession ratio within 13 months of ART initiation. We used linear regression to explore associations between the medication possession ratio and participant characteristics (selected a priori based on findings from previous studies).

#### Qualitative data

For quality control, English and Portuguese transcripts were reviewed by one bilingual investigator (CMA) upon their completion. Two researchers (MPP and CMA) conducted a reflexive thematic analysis using MAXQDA 2020© software [75]. Codes were established, based on Carroll’s conceptual framework for implementation fidelity [74], with a focus on intervention components that were delivered with fidelity (e.g., adherence to education, counseling, and support activities) and adherence to the original protocol. We created codes to highlight activities that were adapted by traditional healers to better fit the local context and identified activities that were not originally included in the intervention but were incorporated into regular activities by the traditional healers. Traditional healer focus groups were conducted first, and participant interviews were used as an opportunity to check comments and concerns raised by the healers. MPP and CMA met to develop, define, and compare application of codes to the transcribed interviews; after which, there was complete agreement of deductive and inductive codes and sub-codes to the 23 interviews and 2 focus groups. These codes were then used to generate the themes in collaboration with JS (a Mozambican expert in integrating traditional healers in the allopathic health system).

## Results

### Characteristics at baseline for all participants

Two hundred seventy-six participants enrolled in HIV care and treatment and agreed to receive support from a traditional healer (Table 1). Immediately after enrollment, participants in the post-intervention group were provided a list (with photos) of trained healers living in their communities and were encouraged to select the person they believed would be the most helpful supporter for them. Overall, the cohort was 53% female with a median (interquartile range, IQR) age of 30 (24–38). The median (IQR) level of education among participants was 3 years (0–6), and three-quarters (78%) of participants were employed in unpaid work (unpaid occupations included farmer, student, or domestic). Two-thirds (68%) of participants were married.

**Table 1:** Participant characteristics at baseline, by group and overall.

	N	Pre *N = 163*	Post *N = 113*	Combined *N = 276*	*P* value
Sex	276				0.66
Female		54% (88)	51% (58)	53% (146)	
Male		46% (75)	49% (55)	47% (130)	
Age	276	30 (23,38)	30 (24,39)	30 (24,38)	0.68
Education Years	276	2 (0,7)	4 (0,6)	3 (0,6)	0.68^2^
Marital Status	276				0.007^1^
Divorced		3% (5)	2% (2)	2% (7)	
Married		70% (114)	65% (74)	68% (188)	
Separated		1% (1)	9% (10)	4% (11)	
Single		17% (28)	12% (13)	15% (41)	
Widow		9% (15)	12% (14)	11% (29)	
Employment	207				0.22^1^
Paid		20% (25)	27% (21)	22% (46)	
Unpaid		80% (103)	73% (58)	78% (161)	

1 Chi-squared test.

2 Rank-sum test.

### Traditional healer characteristics

Forty-two traditional healers volunteered and completed training to provide support to participants. Only 26 were selected by participants. Among all healers, the mean age was 48 years (SD 12.2), most (36, 86%) were married, and only 10 (24%) were female. The mean score for HIV knowledge among healers at baseline was 5.5 (SD 1.96) of a total possible score of 10. Mean narcissism scores were 4.98 (SD 2.02) out of a total possible score of 16, and mean empathy 28.7 (SD 3.2) out of a total possible score of 38. Mean HIV knowledge and empathy scores were slightly higher, and mean narcissism scores lower among healers who were selected by participants (Table 2).

**Table 2:** Healer characteristics at baseline.

	Healers Selected N = 26	Healers Not Selected N = 16
***Demographics***	*N (%)*	*N (%)*
Sex		
Female	5 (19.2)	5 (31.3)
Male	21 (80.8)	11 (68.7)
Age	47.7 ± 13.8	47.6 ± 9.4
Marital Status		
Married	22 (84.6)	14 (87.5)
Single	2 (7.7)	2 (12.5)
Widow	2 (7.7)	0 (0)
***Survey Scores***	*Mean ± SD*	*Mean ± SD*
HIV Knowledge^a^	6.2 ± 1.4	4.5 ± 2.3
Empathy^b^	29.3 ± 3.0	27.7 ± 3.5
Narcissism^c^	2.8 ± 2.2	3.6 ± 1.4

^a^Modeled after the HIV Knowledge 27 Scale; missing HIV knowledge imputed as ‘0’ for incorrect.

^b^Modeled after Jefferson Scale of Empathy; scores range from 0–38; higher scores indicated higher empathy; missing empathy values replaced with mean imputation.

^c^NPI-16 scale; scores range from 0–16; higher scores indicating higher level of narcissism.

#### Predictors of medication possession

The median medication possession ratio was 0.79 (IQR: 0.50–0.91). The medication possession ratio was not significantly different between pre- and post-intervention participants (0.80 in the pre-intervention group compared to 0.79 in the post-intervention group; p = 0.96). Analysis with multivariate linear regression showed that no covariates were significant predictors of our outcome (Table 3). While none of these covariates are significantly predictive of our outcome, we can see that on average, male participants had a 0.074 lower medication possession ratio than female participants, for every year older a participant is, they had a slight increase in their medication possession ratio (0.002), and for every additional year of education a participant had an average increase of 0.007 in their medication possession ratio. Married, separated, single, and widowed participants, on average, all had higher medication possession ratios than those who were divorced.

**Table 3:** Predictors of medication possession among all participants (n = 276).

Variable	Coefficient	95% CI	*P* value
Post-Intervention	0.021	-0.06,0.11	0.62
Sex, Male	-0.074	-0.00,0.01	0.14
Age	0.002	-0.01,0.03	0.22
Education Level	0.007	-0.17,0.43	0.24
Marital Status			
Married	0.126	-0.22,0.68	0.40
Separated	0.230	-0.29,0.34	0.31
Single	0.023	-0.27,0.40	0.88
Widow	0.068	-0.02,0.20	0.68
Unpaid Employment	0.091	-0.06,0.11	0.11

#### Understanding the successes and failures of intervention implementation

While the intervention did not yield a significant improvement in treatment adherence, we conducted interviews with participants and healers to gain insights into what aspects of the program were effective, what failed, and what additional components should be added (if any) to improve the likelihood of success. We conducted qualitative interviews with 23 participants living with HIV who received support from a traditional healer and 19 healers to gain insight into the success and failures of the intervention. Among our 23 participants, 12 were women (52%) with a median age of 35 years (women: 38 years, men 28 years). Healer participants were primarily male (n = 14; 74%) with a median age of 50 years (women: 51 years, men 50 years). All participants were actively engaged in HIV care, although several had gaps in medication pick up during the past year. Healers who participated in our focus group supported a minimum of 4 participants (range: 4–11) during the past year.

#### Why did participants choose to accept a healer’s support?

Participants reported feelings of despair, fear, and overwhelm about the prospect of living with HIV at the time of their diagnosis. When participants were presented with the option of support from a traditional healer, most accepted because they needed help. One participant noted, “I agreed because in that moment I was shaken with the test result, and I truly needed psychological support. In that moment I thought I was going to die quickly.” (Man, 36 years old).

Most participants reported personally knowing, or knowing of, the healer they selected. Participants selected their healer because they already had a comfortable relationship (“He has always been the healer who helped me with traditional medication, he would give me medication even if I didn’t have money to pay.” [Woman, 58 years]) or the healer had a good reputation in the community (“I knew her as a neighbor who worries about the well-being and health of the people in the neighborhood.” [Woman, 30 years old]). Those who did not know anyone on the list picked a healer who lived in their neighborhood to ensure they had access to help if they needed it.

#### What support did participants receive?

*Empathetic education about overcoming medication side effects*. Healers were trained to provide psychosocial counseling, HIV education, and disclosure support to people living with HIV in efforts to increase adherence to treatment. The most common barrier to ART adherence reported by participants was medication side effects, made worse because participants were taking medications on an empty stomach due to extreme poverty. All participants reported experiencing a combination of nausea, vertigo, diarrhea, and headaches. One participant explained:

*I was really bad*! *I was dizzy for one week*. *Then the dizziness went away and I started having diarrhea*. *After the diarrhea I had strong headaches*. *This all happened at the beginning when I started medicating*! *I was almost not taking it anymore*. *But my traditional healer came to talk to me about not stopping*, *that everything I was feeling would go away*. *And it indeed went away and now I have no reactions*. (Woman, 38 years old.)

As trained, healers provided reassurance that the side effects would lessen in time and recommended taking medication with food, however, side effects were made worse due to widespread poverty in the region, where people frequently eat only one meal per day. Healers were able to adapt messages we taught them about food intake with medication to fit the local context more appropriately. Advice about how to navigate side effects in a context of extreme poverty were seen as valuable by participants. One woman explained that her healer “told me that in case I didn’t have dinner, that I should take some sweet potato leaves the next day to cook and eat. He even told me to plant some branches of sweet potato at home.” (Woman, 38 years old). Seventy-five percent of those interviewed reported having to employ this strategy because they did not have food to eat with their medications.

Healers felt an obligation to provide support for those who did not have enough food to take with their daily medication. One healer noted the conflict participants face: “Really, the difficulty that participants face is in feeding. They say, ‘for me, all the advice you give us, we are fulfilling. But we have no food. When I take it without eating, I can’t walk or get out of bed.’” (Traditional Healer, Man, 28 years old.)

Healers feel a responsibility to help, but typically they are also living in poverty and have little to offer. They subsequently had to adapt their solutions due to a lack of available resources. One healer highlighted this conflict:

*The difficulties are the same*: *food is the main factor for people*, *each patient has their own reaction*, *tiredness*, *allergies*, *vomiting*, *nausea*, *drowsiness*, *diarrhea and more*. *And they stay at home without being able to do any activity and so they end up having nothing to eat*. *That’s when we are sorry*, *and we end up taking what little we have to help them*. (Traditional Healer, Man, 27 years old.)

Healers providing food to those they support is unsustainable and unethical and future versions of this intervention will have to address this substantial barrier to adherence.

#### Psychosocial counseling

Healers were trained to provide basic counseling to individuals and couples. Largely, healers felt equipped to provide these services to their participants and felt proud when their participants overcame challenges. Ten participants highlighted the emotional support they received from their healer as a key factor in their remaining in treatment. One woman noted:

…*this program helps people living with HIV to overcome the trauma that they go through after receiving the HIV test result*. *And at the beginning of the HIV treatment*, *when the person gets tested and the result is positive*, *the person gets desperate thinking that her life is over*. *But when they have the support [of someone] accompanying them*, *that person starts to feel better*, *excited to receive advice and realizes that there is more of a chance to live*. (Woman, 32 years old.)

However, healers reported encountering situations they were not equipped to address. While healers were taught counseling strategies to assist in disclosure, there were many aspects of the process that were outside of their control. One described a situation with her patient:

*…whose husband abandoned her*, *she went to her mother’s house*, *and the mother also kicked her out*, *saying she should look for who gave her this disease*. *She went into despair*, *not wanting to live*, *she didn’t want to take the medication because she had nothing to eat*. *I had to feed her every day*. *I was sorry for her situation*. (Traditional Healer, Woman, 51 years old.)

Healers encountered these difficult situations most frequently when a participant’s family was not supportive. Social support systems, including access to food or housing support are essentially non-existent in this region, leaving individuals to manage on their own. Most participants interviewed reported disclosing on their own, but 8 traditional healers spoke about assisting at least 1 participant disclose their HIV status to a family member. One healer explained:

*I had a patient who*, *after taking the test*, *and the result was HIV+ was scared*. *He lived with his brother and was full of fear of revealing about his health to his brother…He wanted to speak*, *but he didn’t know how he was going to do it*. *… He came to talk to me; we went to the brother’s house together and explained everything*. *Thanks to God we had no problems*. (Traditional Healer, Man, 50 years old.)

All participants noted how difficult it would be to take medication without the support of those closest to them. While the healer could help bridge that divide, family members who did not agree with the HIV diagnosis complicated participant efforts to adhere to medication.

#### The adoption of directly observed therapy

Healers reported difficulty in supporting participants believed to be deceptive about ART adherence. In their efforts to address this, and in addition to psychosocial support and advice, healers initiated directly observed therapy (DOT) among participants suspected to be non-adherent, despite this not being taught during training. Borne from a combination of frustration with their participants and experience observing DOT with tuberculosis participants, healers decided that, in many cases, counseling was insufficient. One participant recounted her experience with her healer:

*Every time [the healer] comes over*, *she says that I shouldn’t stop taking [my pills]*, *not fail one day and not miss consultations*. *And there is more*! *Sometimes she asks me for the bottle to check if I’m taking it*, *she counts my pills*, *controls my pick-up day to see if I didn’t fail*, *she does a lot for us*. (Man, 25 years old.)

One healer described how she managed particularly difficult participants:

*I had a patient who had difficulty taking it [ART]*. *When it came time to take it*, *she took the pill out of the bottle and buried it on the floor inside the house*… *I started going to her house every day to give her the pills*. *I’d put it in her mouth*, *give her water*, *check her mouth if she’d swallowed it*, *stayed for a few minutes then I’d leave*. *I did it for (2) months and I was the one who kept the pills in my house*…*and thank God she is now very well and thanked me every day when we see each other*. *She always tells me*, *"if it weren’t for your persistence I would have died already*.*"* (Traditional Healer, woman, 57 years old.)

The use of DOT as a strategy when healers perceived their participants to be deceitful requires additional time and effort, but none of the healers expressed irritation with performing this service. Among participants who experienced this level of observation and control, none complained.

#### Health system challenges in a resource limited setting

The health system is strikingly under-capacitated in rural Mozambique. Nevertheless, our participants only complained about a single issue related to the health system: being made to wait extended periods for medication pickups. One participant explained:

*When we go to the hospital*, *they take a long time to see us*. *When we go in the morning we come back [home] in the afternoon*, *hungry*, *all because we have to go through many doors and each door we have to wait in line*. *So that bothers us a lot*. *(woman 32 years old)Another complained*, *“The hospital service is very delayed*. *When you get there to pick up your medication you become demoralized*, *imagining the distance and the delay*. (Man, 54 years old)

While the patients did not express additional issues with the health system, traditional healers were frustrated that their efforts were not more appreciated by health care workers. Traditional healers saw themselves as working with the system to improve health outcomes but believed some of the workers saw them as barriers to care, or, as unimportant to helping people remain on medication.

*I think that here at the hospital*, *it varies from nurse to nurse*. *For some time now*, *there are days that we are well attended*. *But at the beginning of the program*, *we were very ashamed with the patients*. *Even worse when we passed reference guides [national referral forms from traditional healers to the health system identifying a problem with a patient that needs to be addressed by the health system*, *e*.*g*., *HIV/TB testing*, *malaria treatment]*. *The patient would take three days to be seen*. *Sometimes the patient would come and throw the paper at us*. *I felt very ashamed*. *The patient said*, *“why do you send me to the hospital with this crappy paper*, *which is useless*? *Is it possible for me to go three days without being seen*?*” I wore my [project] t-shirt*, *said let’s go together*, *we arrived at the hospital*, *they did the same thing to us*. *They looked at me as if I was nobody*. *It was too much*, *I was upset*, *the patient too*, *we almost gave up on this job*. *We were told that after dealing with our roots*, *playing drums and then seeing that the patient is not better*, *then take them to the hospital*. *Now what is the problem with nurses*? *This part we didn’t understand the health professionals’ reasons*. *But now it has improved a lot*. *I won’t say that they always treat us well*. *There is still one or another that treat us well*. *There is still one or another that looks at us with contempt and leaves us sitting all day*. (Man, 50 years old)

Another healer responded to this man’s frustration with her own thoughts:

*When we stop to think about it*, *we see that health professionals are not happy with our presence around them*. *In other cases*, *I think they should be happy because we are saving lives just like them*. *Especially because we even go to the pharmacy to pick up pills for the patient*, *check if they are taking at the right time*, *see if they have not missed a day of taking it*, *make constant visits*, *there are those who are in a serious or debilitated state*. *We don’t even have enough time to attend to our traditional side*. (Woman, 57 years)

This lack of camaraderie was disappointing, although we were encouraged by mention of some improvement in healer-health care worker interactions over time. One healer noted:

*Really at the beginning of the program*, *our experience was sad*. *They looked at us and our value*. *But after many struggles in small meetings we had*, *a lot has changed*. *Now I arrive at the hospital*, *I go straight to the service nurse*, *I tell them that I brought a patient*, *it doesn’t take long*, *then they call me to enter*. *They see the patient quickly*, *I go to the pharmacy*, *also soon I’m seen*. (Man, 55 years old)

Future programs will have to continue to facilitate positive contact between these two groups to encourage comradery and support.

## Discussion

Our study has shown that traditional healers can be trained to provide community-based psychosocial support, education, directly observed therapy, and disclosure assistance. Despite positive qualitative reports, healer support did not lead to improvements in patient adherence between the pre- and post-intervention groups.

Researchers have found patient adherence to ART improves when they are provided an essential package of services [76]. These services include provision of: 1) counseling services at testing, treatment initiation, and as needed throughout treatment, 2) clinical services, including immediate initiation of ART [77], monitoring patients for side effects and viral suppression (and adjusting treatment regimens as necessary) [78], 3) treatment for identified clinical complications related to HIV infection [79], and 4) the delivery of all of these services with compassion [80]. Previous studies have found that adherence to treatment and retention to HIV services is significantly higher among people who perceive respect and empathy from providers during appointments, and when providers incorporate the preferences and concerns of people living with HIV into their treatment plan [80]. Our participants suggest that while health care workers may be effectively delivering medications to people living with HIV, they are overwhelmed with providing care in an under capacitated health system. Reports of excessive wait times impacted participants’ desire and ability to remain adherent to treatment. This feedback mirrors results from other studies in rural Mozambique [51,81–83] and throughout sub-Saharan Africa [84–86]. Traditional healers were frustrated that their work was not valued by health care workers and a frustrating finding given the initial work designed to bring the two groups together during the planning phases of this project [51].

Like PLHIV throughout SSA, our participants reported multiple factors that negatively influenced their ability to remain adherent to treatment, including drug side effects, a lack of food, HIV stigma, and a lack of social support [26,36,85,87]. This region of Mozambique is extremely poor, thwarting our participants’ abilities to follow some treatment guidelines, including eating a meal before taking medications [88]. This resulted in vertigo, nausea, and diarrhea among participants. In addition, years of war and internal strife have led to a weak education system, making it difficult to disseminate information about HIV in a way that patients can refer to when necessary [61,89]. Traditional healer strengths included the ability to adapt their education to the local context. While traditional healers were able to help with some of these issues (e.g., suggestions of drinking water with specific types of leaves or tea before taking pills), other social factors (e.g., stigma) were difficult to address.

The experience of our traditional healers reflects that of other community health workers across the continent: the belief that they provide essential education and assistance to people living with HIV but are themselves inadequately supported by the health system [47,90,91]. The traditional healers in our study noted a lack of respect from health care providers, which was surprising given that the health care providers at this facility had been involved in the development and approval of the program [51]. Several studies have tested the ability of healers to refer suspected tuberculosis ([92] and HIV cases[52], and provide support for people living with chronic disease [93–96] with mixed results. While healers may be best positioned to helping people living in the community, systems to further integrate them into the system are necessary to improve patient outcomes.

### Limitations

Our study provides clinical and qualitative data from participants in a pre-post intervention designed to improve HIV treatment adherence. There are several limitations to report. Our study design has inherent problems, including temporal issues due to changes in clinical practice (e.g., changes in treatment eligibility that rolled out in 2018 that allowed for universal test and treat and the elimination of CD4+ cell count collection) that primarily impacted those in the intervention group. This change opened care and treatment to all, suggesting that those in the intervention group may have a higher CD4 cell count (and thus have fewer symptoms related to their HIV disease) than those in the control group. The elimination of routine baseline CD4 cell count collection made it impossible to accurately compare baseline health indicators of participants in each study group. In addition, we used pharmacy pick up data (vs. pill counts or medication levels in urine or blood) to assess adherence. While there is strong evidence that pick-up data is correlated with viral suppression [66,68,69] we may have more precise estimates of impact if we had pill count data from our participants. Lastly, traditional healers are well-established in this region and enjoy a high level of respect from community members. In areas where this is not the case, such an intervention would not be as highly acceptable among people living with HIV.

## Conclusions

Despite seeing no difference in medication coverage among intervention participants, people living with HIV reported positive experiences with traditional healer support and counseling. Healers delivered the intervention with fidelity. However, significant barriers to medication adherence persist. Extreme hunger and fear of stigma, combined with long waits at the health facilities, hindered participants’ desire to adhere to treatment and healers’ ability to support them.

## Supporting information

S1 Checklist (PDF)

S1 Appendix (DOCX)
