# Supplementary material for: “If it weren’t for my traditional healer, I would be dead”: Engaging traditional healers to support people living with HIV in rural Mozambique
Source: PLoS One. 2022 Jun 28;17(6):e0270565. doi: 10.1371/journal.pone.0270565 (PMC9239464; doi:10.1371/journal.pone.0270565)
Supplement: S1 Appendix — (DOCX) [file pone.0270565.s002.docx]

**Appendix 1**

**In-depth Interview Questions for People Living with HIV**

1. Think about the day you received your HIV test results. Do you remember being offered help by a traditional healer? Why did you agree?

2. Can you tell me why you selected ____ (name of healer)? (probes: did you know them? Did you want someone of the same gender?)

3. Can you tell me about your interactions with ______ (name of healer). What kinds of things did you talk about? Can you tell me about the things they taught you?

4. How did they help you with managing your HIV diagnosis? (probe: disclosure assistance, education about symptoms/medication side effects, counseling)

5. Did you want any support that your healer did not provide?

6. How often did you see your healer? Where did you meet them? Would you have preferred to meet more or less frequently?

7. Have you had any difficulties taking your medication every day? How has your healer helped you?

8. Have you had any difficulties with health care workers? How has your healer helped you?

9. Have you revealed your HIV status to your family? How have they reacted to the news? How has your healer helped you with disclosing your status?

10. Thinking about conditions here in _______ village, what additional support could traditional healers provide to people living with HIV?

11. Imagine we were given money to expand the program to other parts of the country. Should we do it? What parts of the program are the most important to keep? Is there anything we should change?

**Focus Group Discussion Guide for Traditional Healers**

1. What kinds of difficulties do people with HIV experience in your community?

2. Why did you decide to work as a treatment support for people living with HIV here in Namacurra?

3. Can you tell me about one of your patients who had the most difficulty remembering to take the medication? What were the main shortcomings they had? How did you help them overcome these difficulties?

4. Can you tell me about one of the patients who was having difficulties picking up the medication at the Health Center? What were the reasons for them not to be able to pick up the medication? How did you help them?

5. Can you tell me about a time when you helped a patient to reveal their HIV+ status to a friend or family member? What advice did you give?

6. How do your patients' families react to learning that their patient is HIV positive? Can you tell me about a time when the family reacted negatively? What happened? (probe: did the husband leave his wife?)

7. Can you tell me when the family reacted positively, what did they do to help?

8. Let's talk about your experience working with people at the Health Center. Have you ever accompanied a patient for an appointment? What kinds of things did you talk to health professionals about? How were you treated by the health team?

9. Let's talk about the training you received to help people living with HIV. What information did you find useful? What additional training would you like to have?

10. Imagine that we expand this project in the province of Zambézia, even in the whole country. There are many patients and very few healers to support everyone each year. Were we thinking that maybe healers could only visit their patients for the first 3 months, or the first 6 months? When is the best, most important time when a patient needs help or support?

11. Do you have any other suggestions to improve the program?
